# Fullerene-modified magnetic silver phosphate (Ag_3_PO_4_/Fe_3_O_4_/C_60_) nanocomposites: hydrothermal synthesis, characterization and study of photocatalytic, catalytic and antibacterial activities

**DOI:** 10.1039/c8ra00069g

**Published:** 2018-03-13

**Authors:** Shahnaz Sepahvand, Saeed Farhadi

**Affiliations:** Department of Chemistry, Lorestan University Khorramabad 68151-44316 Iran sfarhadi1348@yahoo.com +98-6633120611 +98-6633120618

## Abstract

In this work, fullerene-modified magnetic silver phosphate (Ag_3_PO_4_/Fe_3_O_4_/C_60_) nanocomposites with efficient visible light photocatalytic and catalytic activity were fabricated by a simple hydrothermal approach. The composition and structure of the obtained new magnetically recyclable ternary nanocomposites were completely characterized by X-ray diffraction (XRD), Fourier transform infrared spectroscopy (FT-IR), Raman spectroscopy, Brunauer–Emmett–Teller (BET) specific surface area analysis, vibrating sample magnetometery (VSM), diffuse reflectance spectroscopy (DRS), field emission scanning electron microscopy (FE-SEM), energy dispersive X-ray (EDX) spectroscopy and transmission electron microscopy (TEM). This novel magnetically recyclable heterogeneous fullerene-modified catalyst was tested for the H_2_O_2_-assisted photocatalytic degradation of MB dye under visible light. The results show that about 95% of the MB (25 mg L^−1^, 50 ml) was degraded by the Ag_3_PO_4_/Fe_3_O_4_/C_60_ nanocomposite within 5 h under visible light irradiation. The catalytic performance of the Ag_3_PO_4_/Fe_3_O_4_/C_60_ nanocomposite was then examined for 4-nitrophenol (4-NP) reduction using NaBH_4_. This new nanocomposite showed that 4-NP was reduced to 4-aminophenol (4-AP) in 98% yield with an aqueous solution of NaBH_4_. In both photocatalytic and catalytic reactions, the Ag_3_PO_4_/Fe_3_O_4_/C_60_ nanocomposite exhibited higher catalytic activity than pure Ag_3_PO_4_. Moreover, the Ag_3_PO_4_/Fe_3_O_4_/C_60_ nanocomposite could be magnetically separated from the reaction mixture and reused without any change in structure. The antibacterial activity of the nanocomposites was also investigated and they showed good antibacterial activity against a few human pathogenic bacteria.

## Abstract 2

Fullerene-modified magnetic silver phosphate (Ag_3_PO_4_/Fe_3_O_4_/C_60_) nanocomposites prepared by a hydrothermal route were used as photocatalysts/catalysts for the efficient degradation and reduction of MB dye and 4-nitrophenol, respectively.

## Graphical abstract

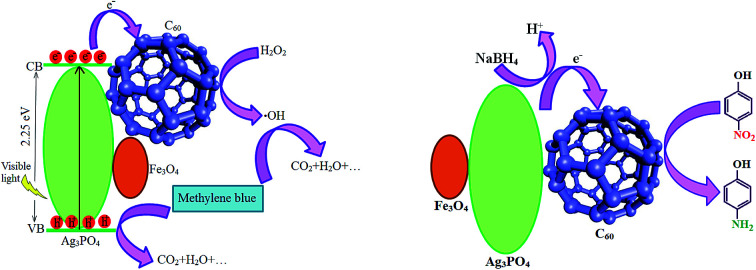

## 1. Introduction

Today, water pollution is one of the main problems that human beings encounter. Every day, human activities lead to the release of contaminant substances and waste into the rivers, lakes, groundwater aquifers and oceans. This contamination affects the aquatic environmental quality for various uses and human consumption.^1^ Water pollutants, including organic material such as methyl orange, methylene blue and rhodamine B dyes, are hazardous, toxic and carcinogenic for humans even at low concentrations, and they are hardly biodegradable and difficult to remove from the environment.^2^ The ingestion of liquid products containing concentrated nitrophenol can cause serious gastrointestinal damage and even death. In animals, longer-term exposure to high levels of nitrophenol cause damages to the heart, kidneys, liver and lungs. It is therefore very important to find innovative and cost-effective ways for the complete removal of organic pollutants and for monitoring water safety.^3,4^ One way is the production of catalysts to eliminate water contamination, either in the dark or in visible light. Certainly, due to their high efficiency and promising economy, semiconductor-based catalytic and photocatalytic technologies have opened up new opportunities to control pollutants and deal with their effects.^5–10^ To date, various metal oxides, sulfides, carbon compounds and composite materials have been investigated for the development of effective photocatalysts.^11–15^

In recent years, considerable attention has been paid to silver orthophosphate (Ag_3_PO_4_), a new photocatalyst with an extremely high photooxidative capability for O_2_ generation from water splitting. This is due to its highly positive VB position, low toxicity and superior photodegradation rate of organic dyes, which is dozens of times faster than the surface level of commercial TiO_2_ under visible light irradiation.^16–24^ Unfortunately, because of its low structural stability, it is possible for Ag_3_PO_4_ to be photochemically decomposed to Ag if no sacrificial reagent is involved.^25^ Increasing both the stability and the catalytic activity of pure Ag_3_PO_4_ by coupling Ag_3_PO_4_ with other materials has been proven to be an effective strategy.^26^ Many Ag_3_PO_4_-based composites, such as Ag_3_PO_4_/CeO_2_,^27^ Ag_3_PO_4_/Fe_3_O_4_/GO,^28^ AgX/Ag_3_PO_4_ (X = Cl, Br, I),^29^ Ag_3_PO_4_/TiO_2_,^30^ CdS/Ag_3_PO_4_,^31^ Ag_3_PO_4_/MoS_2_/GR,^32^ Ag_3_PO_4_/Bi_2_WO_6_,^33^ Ag_3_PO_4_/BiVO_4_,^34^ Ag_3_PO_4_/LaFeO_3_,^35^ Ag_3_PO_4_/Co_3_O_4_,^36^ Ag_3_PO_4_/CoFe_2_O_4_,^37^ Ag_3_PO_4_/AgI/MWCNTs,^38^ TiO_2_/Ag_3_PO_4_/GR,^39^ P_25_/Ag_3_PO_4_/GO,^40^ g-C_3_N_4_ nanorod/Ag_3_PO_4_ (ref. 41) and GO/Ag_3_PO_4_,^42^ display enhanced stability and photocatalytic activity.

Carbon materials have potential applications in many fields of environmental pollution control due to their special properties, such as their higher specific surface area, superior electronic characteristics, confinement effects and strong physical/chemical stability.^43–46^ Among the large family of carbon-based nanomaterials, fullerene (C_60_) is known as an excellent electron acceptor with an appropriate band gap of 1.7–1.9 eV, and thus can lead to rapid photoinduced charge separation and relatively slow charge recombination.^47,48^ A C_60_ molecule has a closed shell configuration consisting of 30 bonding molecular orbitals with 60 p electrons, which is advantageous for shuttling and transporting electrons.^49,50^ Moreover, this unique structure endows C_60_ with many other intriguing characteristics, which mainly include its excellent exciton mobility, high thermal stability, low density, strain-tunable semiconducting characteristics, moderate elastic modulus and high bending flexibility.^51,52^ From the perspective of the excellent properties of C_60_, hybridizing C_60_ with Ag_3_PO_4_ should be beneficial for improving the photocatalytic performance of Ag_3_PO_4_. However, separation and recycling of the binary Ag_3_PO_4_/C_60_ composite is difficult and severely limits its potential applications. To overcome this problem, coupling with magnetic materials *e.g.* Fe_3_O_4_ is highly desirable. Such magnetic composite catalysts can be easily recovered by a magnet and reused for catalytic reactions several times without any considerable reduction in catalytic efficiency.

In the present work, we explored the role of C_60_ towards the photochemical performance of hybridized Ag_3_PO_4_/Fe_3_O_4_/C_60_ composites. The structural characteristics of Ag_3_PO_4_/Fe_3_O_4_/C_60_ with a varied C_60_ content were first studied, then their performances were evaluated by the visible light photocatalytic degradation of MB dye and the catalytic reduction of 4-nitrophenol (4-NP). Based on the characterization and photocatalytic/catalytic results, possible mechanisms were proposed. The results of this work demonstrate that hybridizing C_60_ with Ag_3_PO_4_ could improve the separating efficiency of photoinduced electrons and holes, which resulted in the enhanced photocatalytic activity of Ag_3_PO_4_/Fe_3_O_4_/C_60_ composites. The antibacterial activity of the nanocomposites was also investigated.

## 2. Experimental

### 2.1. Materials

All chemicals were reagent grade and were used without further purification, such as iron(ii) diammonium sulfate hexahydrate ((NH_4_)_2_FeSO_4_·6H_2_O), iron(iii) ammonium bisulfate dodecahydrate (NH_4_Fe(SO_4_)_2_·12H_2_O), sodium borohydride (NaBH_4_), absolute ethanol, disodium hydrogen phosphate (Na_2_HPO_4_), silver nitrate (AgNO_3_), 4-nitrophenol, 2-nitrophenol, 4-nitroaniline, 2-nitroaniline, hydrogen peroxide (H_2_O_2_, 30%) and methylene blue (MB, C_16_H_18_ClN_3_S). The reagents were purchased from Merck and used as received. Fullerene (C_60_, 99.9%) was purchased from Sigma-Aldrich. Double distilled deionized water was used for the experiments. All glassware was properly washed with distilled water and dried in an oven.

### 2.2. Synthesis of the Ag_3_PO_4_/Fe_3_O_4_/C_60_ (m-APO/C_60_) nanocomposites

The Fe_3_O_4_ nanoparticles were prepared through a hydrothermal process. 1 mmol Fe^2+^ and 2 mmol Fe^3+^ were dissolved in 30 ml deionized water and an appropriate amount of NaOH was added, and the pH was set to be 11 at 50 °C for 10 min with continuous stirring, yielding a uniform black suspension. It was then transferred to an autoclave (50 ml) at 180 °C for 20 h. Subsequently, the autoclave was cooled to room temperature naturally. The as-obtained black samples were centrifuged, washed with deionized water and ethanol three times, and dried at 70 °C for 3 h. To prepare the Ag_3_PO_4_/Fe_3_O_4_/C_60_ (wt 5%) nanocomposite, a mixture of 0.2 g of the Fe_3_O_4_ nanoparticles dispersed in 5 ml deionization water, 1 mmol of Na_2_HPO_4_·12H_2_O, 3 mmol of AgNO_3_ and 10 ml of C_60_ toluene solution (1 g L^−1^) were stirred for 30 min. After sonication for 30 min, the homogenized suspension was transferred into a 50 ml Teflon-lined stainless steel autoclave, sealed and maintained at 180 °C for 20 h. The autoclave was then naturally cooled to room temperature and the resulting precipitate was separated by a magnet, washed with deionized water several times, dried at 60 °C and used for further characterization. It was then transferred to a 50 ml autoclave and heated at 180 °C for 20 h. Subsequently, the autoclave was cooled to room temperature naturally. The product was collected by applying an external magnetic field, washed several times with absolute ethanol and distilled water, and finally dried at 70 °C for 3 h. The samples with 10 and 20 wt% of C_60_ were prepared in a similar manner. For comparison, the pure Ag_3_PO_4_ nanostructure was synthesized according to the typical synthesis described above with Fe_3_O_4_ and C_60_ being absent. The obtained samples with 5, 10 and 20 wt% of C_60_ and pure Ag_3_PO_4_ are denoted as m-APO/C_60_(5), m-APO/C_60_(10), m-APO/C_60_(20) and APO, respectively.

### 2.3. Photocatalytic dye degradation tests

Photocatalytic degradation of the aqueous solution of methylene blue (MB) was carried out in the presence of the m-APO/C_60_ photocatalyst using a 400 W high pressure mercury lamp as an irradiation source, with a cool water circulating filter to absorb the near IR and a UV light cut-off filter to avoid direct photolysis of the organic dyes (*λ* ≥ 420 nm). In a typical experiment, 0.05 g of the m-APO/C_60_ photocatalyst was added to 50 ml of MB (25 mg L^−1^) to perform the photocatalytic degradation. Before irradiation, the solution was stirred for 30 min to achieve an adsorption–desorption equilibrium of the dye on the photocatalyst surface. It was then subjected to visible light irradiation in the presence of H_2_O_2_. At given time intervals, 2 ml aliquots of the reaction solution were sampled, and the catalyst was immediately separated from the suspension by an external magnetic field. The residual MB concentration was determined using a UV-Vis spectrophotometer.

### 2.4. Catalytic reduction tests

In order to explore the catalytic performance of the synthesized m-APO/C_60_ nanocomposites, the reduction of 4-nitrophenol (4-NP) to 4-aminophenol (4-AP) by sodium borohydride (NaBH_4_) in aqueous solution was used as the model reaction. In a typical catalytic reaction, 3 ml of an aqueous solution of 4-NP (0.2 mM) and 0.7 ml of an aqueous solution of NaBH_4_ (20 mM) were mixed in a standard quartz cell with a 1 cm path length, then 2.5 mg of the synthesized nanocomposite was added to the reaction mixture. Immediately afterwards, the catalysts were transferred to a standard quartz cell while the nitrophenol concentration in the reaction mixture was monitored by UV-visible absorption spectra, recorded with a time interval of 2 min in a scanning range of 200–800 nm at ambient temperature. After the completion of the reaction, in order to perform the recycling experiment, the catalyst was recovered first by an external magnet and then by centrifugation. The precipitate was washed repeatedly with deionized water and absolute ethanol in consecutive washing cycles. After washing and placing in a furnace in order to remove adsorbed impurities, the catalyst was used directly for the recycling test. After each cycle, the resulting catalyst was collected and detected by atomic absorption spectroscopy to determine the content of the synthesized nanocomposites.

### 2.5. Antibacterial tests

The antibacterial activity of the synthesized nanoparticles was evaluated against strains of Gram-positive bacteria (*Bacillus cereus* (PTCC 1015) and *Staphylococcus aureus* (1431)) and Gram-negative bacteria (*Escherichia coli* (PTCC 1330) and *Klebsiella pneumoniae* (PTCC 1290)) using a modified Kirby–Bauer disk diffusion method.^53^ Bacteria were cultured for 18 h at 37 °C in a nutrient agar medium and then adjusted with sterile saline to a concentration of 1 × 10^6^ cfu ml^−1^. Bacterial suspensions in Petri dishes (8 cm) containing sterile Mueller-Hinton agar (MA) were cultured using a sterile cotton swab. The compounds were dissolved in water and sterile paper discs of 6 mm thickness were saturated with 30 μl of the samples and placed onto agar plates which had previously been immunized with the tested microorganisms. Amikacin (30 μg per disk) for Gram-negative and penicillin (10 μg per disk) for Gram-positive were used as positive controls. After incubation at 37 °C for 24 h, the inhibition zone diameter was measured using a meter ruler and the mean value for each organism was recorded and expressed in millimeters.

### 2.6. Materials characterization

FT-IR spectra were recorded on a Shimadzu FT-IR 8400S spectrophotometer in transmission mode from 4000 to 400 cm^−1^ using KBr pellets. The XRD patterns of the samples were obtained on an X-ray diffractometer (Rigaku D/Max C III) using Ni-filtered Cu Kα radiation (*λ* = 1.5406 Å). UV-Vis diffuse reflection spectroscopy (DRS) was performed on a Snico S4100 spectrophotometer over the spectral range of 200–1000 nm using BaSO_4_ as the reference. The shapes and morphologies of the samples were observed by a MIRA3 TESCAN field emission scanning electron microscope (FESEM) equipped with a link energy-dispersive X-ray (EDX) analyzer. The particle size was determined by a CM120 transmission electron microscope (TEM) at an accelerating voltage of 80 kV. TEM samples were prepared by dropping the ethanol dispersion onto a carbon coated copper grid. A PHS-1020 PHSCHINA instrument was used to measure the Brunauer–Emmett–Teller (BET) surface areas of the samples at liquid nitrogen temperature (77 K). Magnetic measurements were carried out at room temperature using a vibrating sample magnetometer (VSM, Magnetic Daneshpajoh Kashan Co., Iran) with a maximum magnetic field of 10 kOe. Raman spectroscopy was performed using a SENTERRA (2009) dispersive Raman microscope from BRUKER (Germany) with a laser wavenumber of 785 nm. UV-Vis spectra of the aqueous solutions during the reaction were recorded using a Cary 100 double beam spectrophotometer operated at a resolution of 2 nm using quartz cells with path length of 1 cm.

## 3. Results and discussion

### 3.1. Characterization of the Ag_3_PO_4_/Fe_3_O_4_/C_60_ (m-APO/C_60_) nanocomposites

**Fig. 1:**
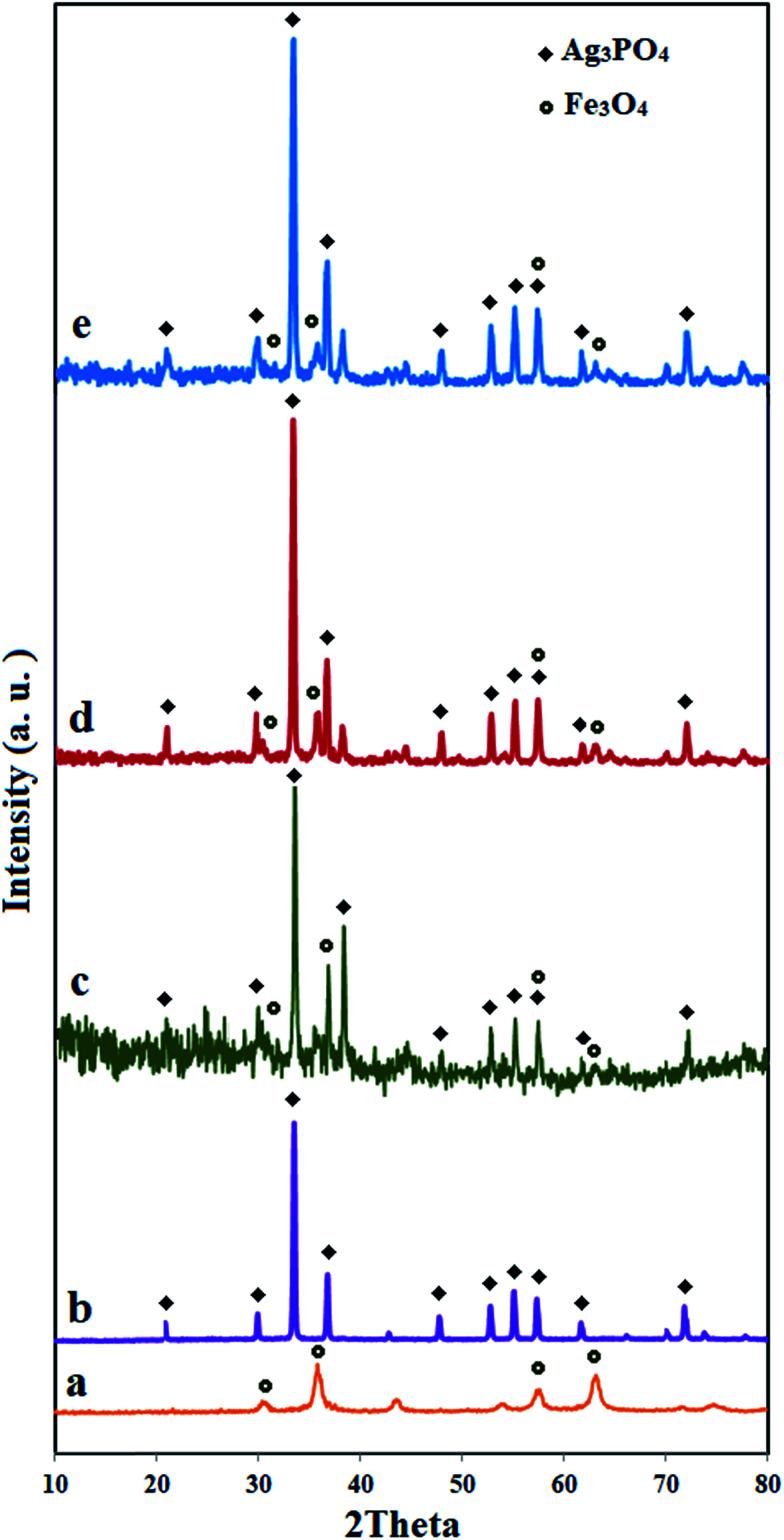
XRD patterns of (a) Fe_3_O_4_, (b) APO, (c) m-APO/C_60_(5), (d) m-APO/C_60_(10) and (e) m-APO/C_60_(20).

The composition and crystal structures of the as-prepared samples were investigated by XRD, as shown in Fig. 1. Ag_3_PO_4_, Ag_3_PO_4_/Fe_3_O_4_/C_60_ (wt 5%), Ag_3_PO_4_/Fe_3_O_4_/C_60_ (wt 10%) and Ag_3_PO_4_/Fe_3_O_4_/C_60_ (wt 20%) are abbreviated as APO, m-APO/C_60_(5), m-APO/C_60_(10) and m-APO/C_60_(20), respectively. In the as-prepared m-APO/C_60_ samples, no other impurities could be observed. It was found that the as-prepared catalysts exhibited intense and sharp diffraction peaks ascribed to cubic crystal systems for Ag_3_PO_4_ (JCPDS card no. 84-0511) and Fe_3_O_4_ (JCPDS card no. 75-0449). No diffraction peaks corresponding to C_60_ were observed in the m-APO/C_60_ nanocomposites, which may be due to the relatively low diffraction intensity of C_60_ and the high dispersion of the small amount of C_60_ in the sample. The average grain sizes of the as-prepared m-APO/C_60_(5), m-APO/C_60_(10) and m-APO/C_60_(20) nanocomposites were calculated to be 31.75 nm, 27.76 nm and 24.86 nm, respectively, based on the Debye–Scherrer formula:^54^*D*_XRD_ = 0.9*λ*/(*β* cos *θ*), where *D*_XRD_ is the average crystallite size, *λ* is the wavelength of Cu Kα radiation, *β* is the full-width at half-maximum of the chosen diffraction peak and *θ* is the Bragg angle.

**Fig. 2:**
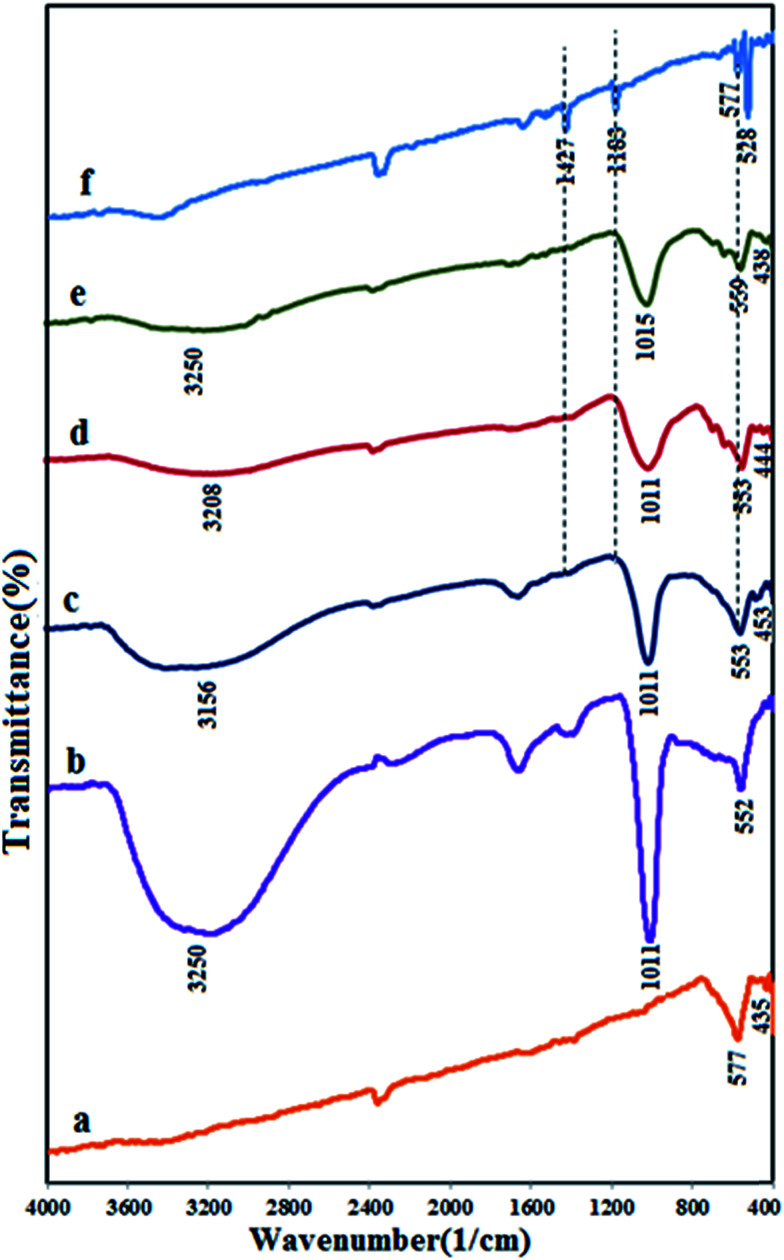
FT-IR spectra of (a) Fe_3_O_4_, (b) APO, (c) m-APO/C_60_(5), (d) m-APO/C_60_(10), (e) m-APO/C_60_(20) and (f) pure C_60_.

Fig. 2 shows the FT-IR spectra of Fe_3_O_4_, APO, m-APO/C_60_(5), m-APO/C_60_(10), m-APO/C_60_(20) and pure C_60_. In the case of Fe_3_O_4_ (Fig. 2(a)), the two peaks in the range of 400 to 600 cm^−1^ are related to the Fe(iii)–O and Fe(ii)–O bond of spinel-type Fe_3_O_4_ oxide. For the Ag_3_PO_4_ sample in Fig. 2(b), the sharp peaks at *ca.* 1011 and 552 cm^−1^ are the characteristic stretching and bending vibrations of PO_4_^3−^ groups, respectively. The FT-IR spectra of the m-APO/C_60_ samples in Fig. 2(c)–(e) show the representative PO_4_^3−^ group stretching vibration mode at 1011 cm^−1^ as well as the featured absorption bands of Fe_3_O_4_ in the 400–600 cm^−1^ range. These findings confirm the coexistence of Ag_3_PO_4_ and Fe_3_O_4_ in the m-APO/C_60_ nanocomposites, in agreement with the XRD results. In all samples, the peaks at 1615 and 3250 cm^−1^ are attributed to the typical stretching vibrations of the –OH group of adsorbed water. In addition, the FT-IR spectrum of the pure C_60_ sample in Fig. 2(f) showed weak peaks at 1183 and 1427 cm^−1^, ascribed to the internal modes of C_60_.^55^ Therefore, the characteristic weak bands of the C_60_ component in the m-APO/C_60_ nanocomposites are hardly observed. To confirm the strong combination between the C_60_ and APO/Fe_3_O_4_ nanoparticles, we also investigated whether C_60_ can leach from the m-APO/C_60_ hybrid system in toluene solution. The color of the toluene solution turned purple after adding C_60_ and sonicating at room temperature for 15 min. However, no color change was observed when using the as-obtained m-APO/C_60_ composites under the same conditions. These results clearly show that the loaded C_60_ clusters in the hybrid system could not be extracted by an excellent solvent (toluene), suggesting the formation of a strong interaction between the C_60_ clusters and APO/Fe_3_O_4_.

**Fig. 3:**
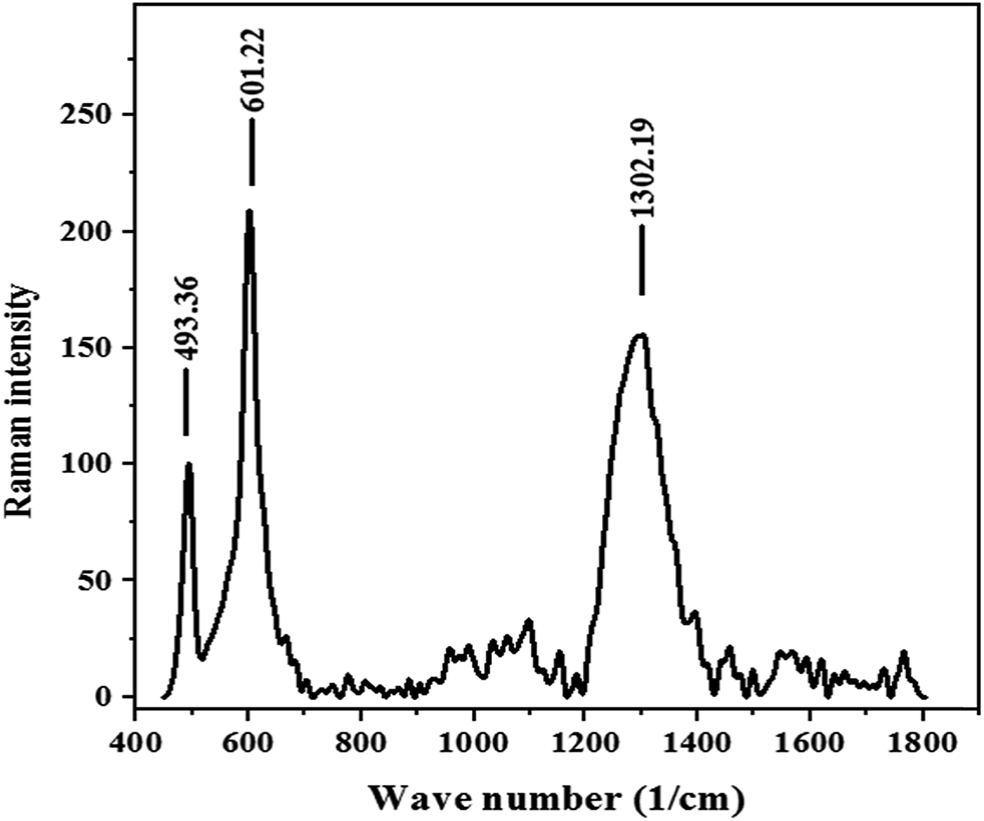
Raman spectrum of m-APO/C_60_(5).

Raman spectroscopy is a powerful nondestructive tool to characterize the significant structural changes in the carbon nanostructures during nanocomposite synthesis. Fig. 3 shows the Raman spectrum of the m-APO/C_60_(5) sample. This sample displays a characteristic C_60_ band at 1302 cm^−1^, as well as the characteristic bands of Ag_3_PO_4_ and Fe_3_O_4_ at 601 and 493 cm^−1^, respectively.^56^ Thus, this observation confirms the presence of C_60_ molecules in the nanocomposites.

**Fig. 4:**
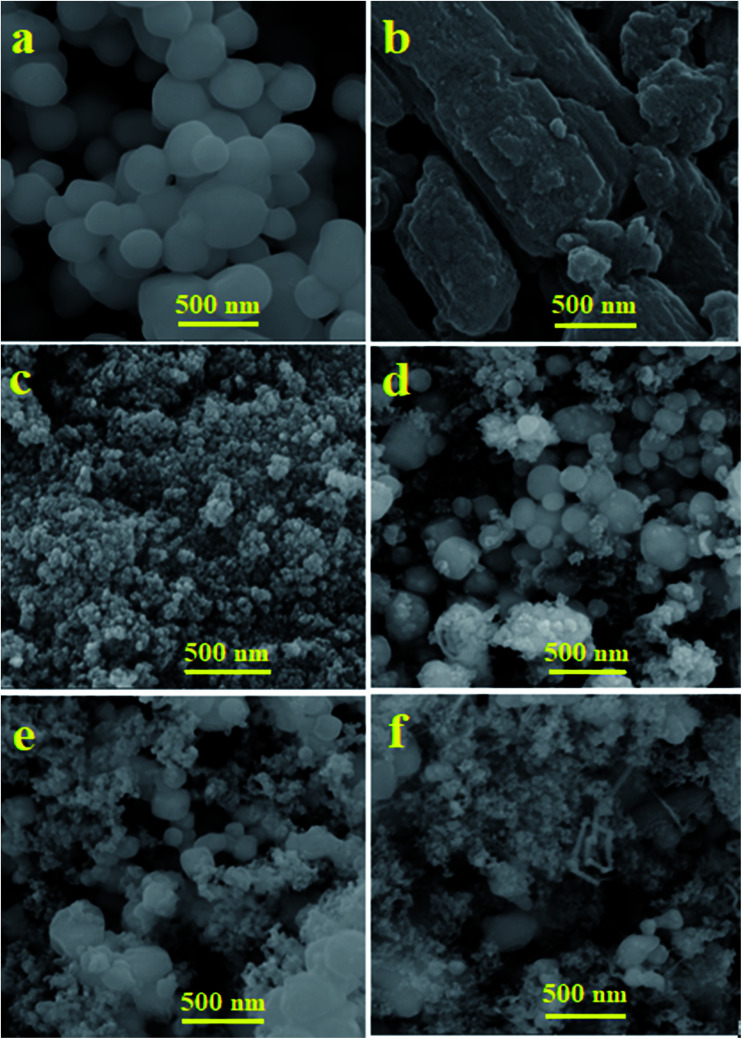
FE-SEM images of (a) APO, (b) C_60_, (c) Fe_3_O_4_, (d) m-APO/C_60_(5), (e) m-APO/C_60_(10) and (f) m-APO/C_60_(20).

SEM images showing the morphologies and microstructural features of Ag_3_PO_4_, C_60_, Fe_3_O_4_ and the m-APO/C_60_(5) nanocomposite are shown in Fig. 4. The SEM images in Fig. 4 demonstrate the morphologies of pure Ag_3_PO_4_ C_60_, Fe_3_O_4_ and the m-APO/C_60_(5) nanocomposite. As shown in Fig. 4(a), the SEM image of Ag_3_PO_4_ consists of near spherical particles with diameters in the range of 50–100 nm, which are slightly agglomerated. Fig. 4(b) shows the characteristic morphology of C_60_ and Fig. 4(c) shows that pure Fe_3_O_4_ consists of small, fine nanoparticles with sizes of 20–30 nm. From Fig. 4(d), it can be seen that the m-APO/C_60_(5) nanocomposite still retains the spherical morphology of Ag_3_PO_4_, and the distribution of C_60_ and Fe_3_O_4_ nanoparticles in the Ag_3_PO_4_ matrix is homogeneous. Compared to Ag_3_PO_4_, m-APO/C_60_ is more scattered. Fig. 4(e) and (f) are SEM images of the m-APO/C_60_ nanocomposites with 10 and 20 wt% of C_60_. The particle size of the m-APO/C_60_ nanocomposites decreased slightly after modifying with C_60_. This may be due to the presence of C_60_ between the m-Ag_3_PO_4_/Fe_3_O_4_ nanoparticles. SEM images of the m-APO/C_60_ nanocomposites with different amounts of C_60_ indicated that the presence of C_60_ had a large effect on the size of the APO nanoparticles, and as the size of particles became smaller, the amount of C_60_ increased.

**Fig. 5:**
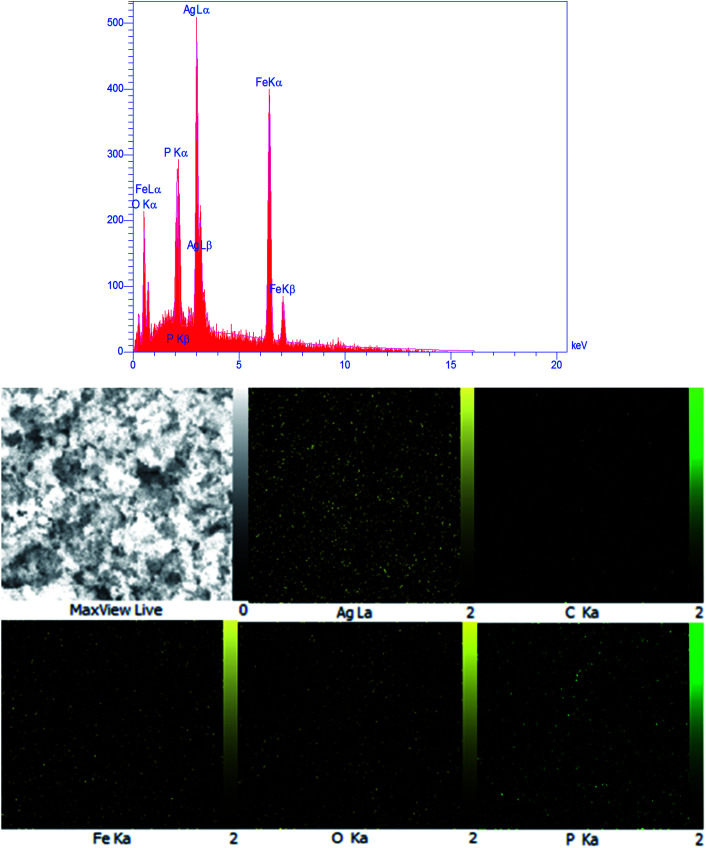
EDX spectrum and elemental mapping of the m-APO/C_60_(5) nanocomposite.

The composition of the as-prepared m-APO/C_60_(5) composites was further investigated by EDX analysis. Fig. 5 shows the EDX spectrum and a representative SEM image of m-APO/C_60_(5) with the corresponding EDX elemental mappings. The presence of Ag, C, Fe, O and P elements can be confirmed by their peaks in the EDX elemental spectrum. The corresponding elemental mappings show that the Ag, C, Fe, O, and P elements are uniformly distributed over the nanocomposite, confirming the homogeneity of the sample. The Fe and C elements were from Fe_3_O_4_ and C_60_, and the results further indicate that the Fe_3_O_4_ and C_60_ particles were successfully coupled with APO.

**Fig. 6:**
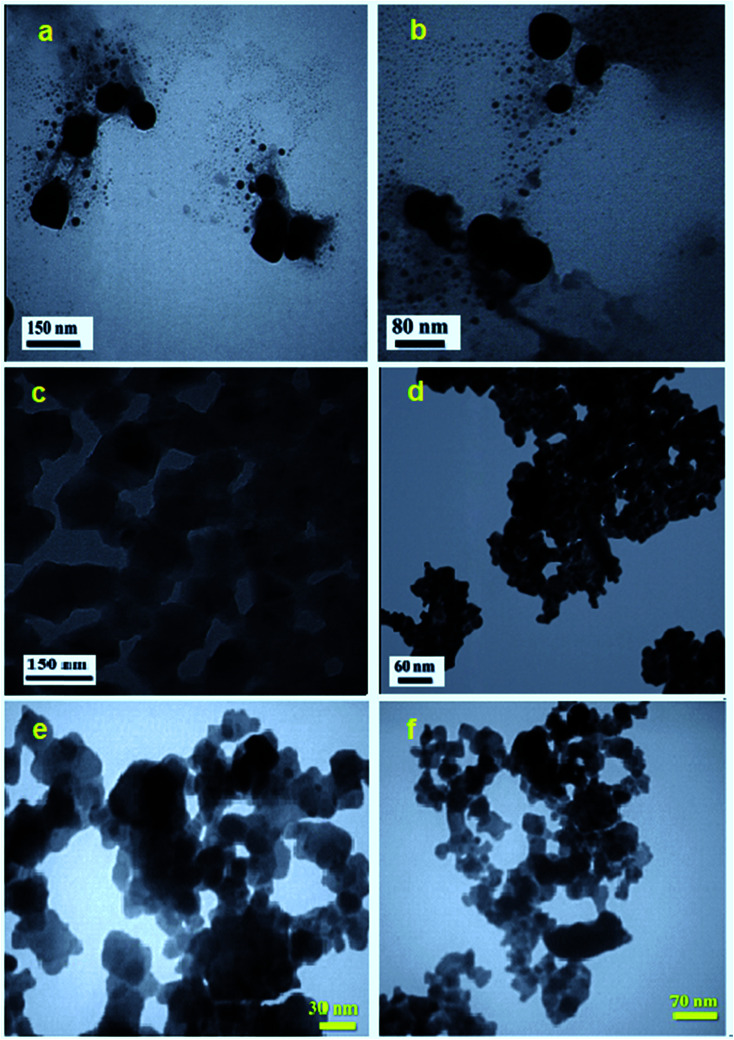
TEM images of (a and b) pure APO, (c) C_60_, (d) Fe_3_O_4_ and (e and f) m-APO/C_60_(5).

The shape and particle size of the as-synthesized APO, C_60_, Fe_3_O_4_ and m-APO/C_60_(5) samples were further investigated by TEM, and the results are shown in Fig. 6. The TEM images of the Ag_3_PO_4_ sample in Fig. 6(a) and (b) show nearly uniform monodispersed spheres with an average diameter of about 50–150 nm. The SEM image in Fig. 6(c) shows that the bare C_60_ sample was formed from plate-like particles which were loosely aggregated. From the TEM image in Fig. 6(d), it was found that pure Fe_3_O_4_ was formed of very fine and agglomerated sphere-like nanoparticles with an average diameter of about 20 nm. The accumulation of these nanostructures can be attributed to the magnetic dipole interaction between them and the great surface energy due to their nanoscopic size. TEM images of the m-APO/C_60_(5) nanocomposite are shown in Fig. 6(e) and (f). By comparing with the SEM images, it is evident that the shape and morphology of the m-APO/C_60_(5) particles are similar to those of the pure components. The morphology of the prepared nanocomposite was sphere-like (APO/C_60_(5)) and plate-like (C_60_), and the particle size distribution was narrow (from ∼20 nm to ∼40 nm) with an average particle size of 30 nm. From the images, it can be clearly seen that many spherical APO and Fe_3_O_4_ particles with sizes of about 15–20 nm were well-coupled with the C_60_ nanoplates. It is reasonable to assume that C_60_ clusters were successfully incorporated into the m-APO/C_60_ samples while retaining their special structures.

**Fig. 7:**
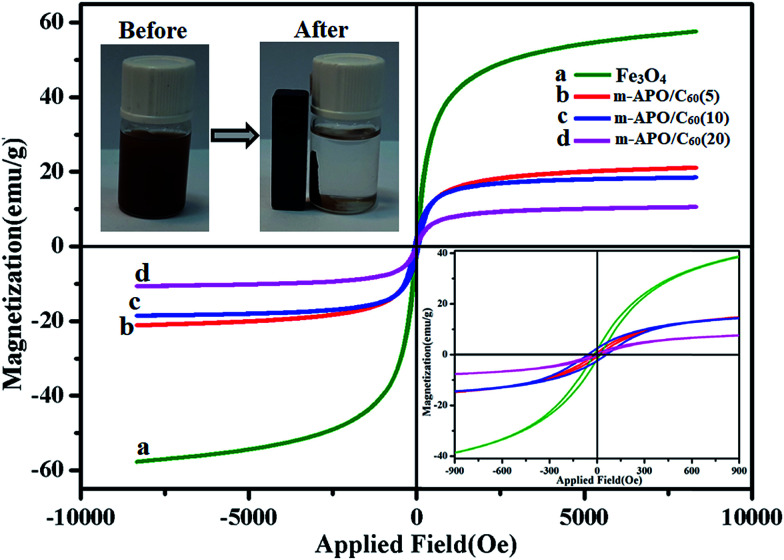
Magnetic hysteresis loops of (a) pure Fe_3_O_4_, (b) m-APO/C_60_(5), (c) m-APO/C_60_(10) and (d) m-APO/C_60_(20). The inset shows the magnetic separation of the m-APO/C_60_(5) nanocomposite from aqueous solution by an external magnet.

The magnetic behavior of the pure Fe_3_O_4_ and m-APO/C_60_ nanocomposites was investigated by VSM at room temperature. The magnetization curves of the prepared nanocomposites of the m-APO/C_60_(5), m-APO/C_60_(10) and m-APO/C_60_(20) nanocomposites and pure Fe_3_O_4_ are shown in Fig. 7. Increasing the applied field from −10 000 to 10 000 Oe caused the magnetization to undergo a sharp increase. At this point, the magnetization was saturated at about 8500 Oe for the prepared nanocomposite. By comparing the magnetic remnant (*M*_r_) and coercivity field (*H*_c_) values of the pure Fe_3_O_4_ sample and the m-APO/C_60_ nanocomposites in Table 1, it can be understood that the m-APO/C_60_(5) and m-APO/C_60_(20) nanocomposites had superparamagnetic properties while the m-APO/C_60_(10) nanocomposite had ferromagnetic properties, compared to Fe_3_O_4_, which had values of *M*_r_ = 3.68 emu g^−1^ and *H*_c_ = 31.46 Oe, showing strong ferromagnetic properties. All catalysts were collected in the inner flank of the beaker after 30 s in the presence of an external magnetic field (see the inset of Fig. 7). The results confirmed the high magnetization as well as the extremely high reusability of the m-APO/C_60_ nanocomposites, implying their high potential and promising applications for water purification to avoid secondary pollution.

**Table 1:** Magnetic properties of the pure Fe_3_O_4_ sample and the m-APO/C_60_ nanocomposites

Sample	Magnetic remnant (*M*_r_) (emu g^−1^)	Magnetic saturation (*M*_s_) (emu g^−1^)	Coercivity field (*H*_c_) (Oe)
Fe_3_O_4_	3.68	57.84	31.46
m-APO/C_60_(5)	0.76	21.15	30.28
m-APO/C_60_(10)	3.13	18.55	60.69
m-APO/C_60_(20)	0.58	10.59	50

**Fig. 8:**
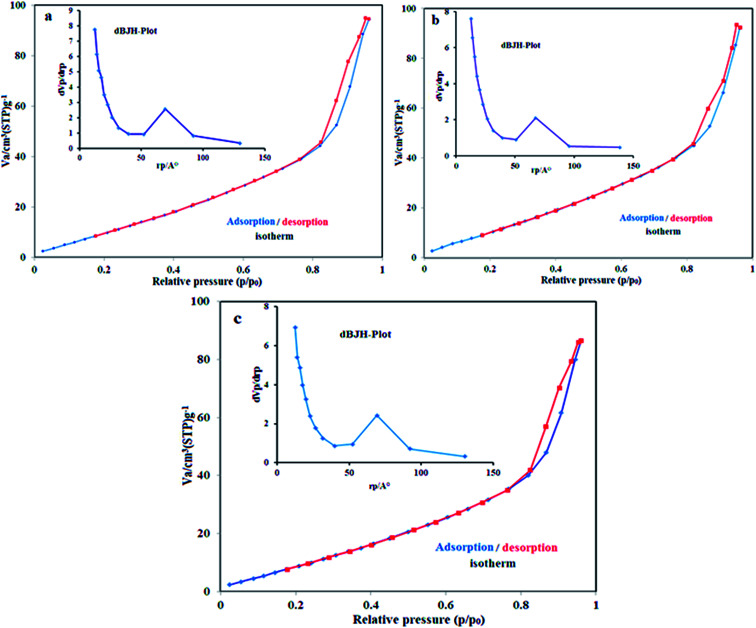
N_2_ adsorption–desorption isotherms of (a) m-APO/C_60_(5), (b) m-APO/C_60_(10) and (c) m-APO/C_60_(20). The insets show the corresponding pore size distribution curves.

Nitrogen adsorption–desorption experiments were used to evaluate the pore size and structure of the samples. The porosity and specific surface area were determined using the Barrett–Joyner–Halenda (BJH) method and the BET equation, respectively.^57,58^ The calculated BET specific surface area of the m-APO/C_60_(5) nanocomposite was 55.461 m^2^ g^−1^ (see Fig. 8). The pore size and pore volume distributions of the m-APO/C_60_(5) nanocomposites were centered at 1.26 nm and 0.212 cm^3^ g^−1^, respectively. The isotherm in Fig. 8(a)–(c) can be classified as type IV with a H_4_ hysteresis loop for the m-APO/C_60_(5), m-APO/C_60_(10) and m-APO/C_60_(20) nanocomposites. The BET surface area values for the m-APO/C_60_(5), m-APO/C_60_(10) and m-APO/C_60_(20) nanocomposites were higher than those for pure APO and m-APO (Table 2).^59^ It can be concluded that the addition of Fe_3_O_4_ and C_60_ to the APO significantly affected the microstructure of APO and greatly increased the surface area and pore volume, all of which were considered to be favorable factors for the improvement of the photocatalytic performance. In comparison to pure APO, the microporous structure and relatively high surface area of the prepared m-APO/C_60_(5) nanocomposites were expected to have higher photocatalytic activity.

**Table 2:** Textural properties of the pure APO and m-APO/C_60_ samples^a^

Samples	*S*_BET_ (m^2^ g^−1^)	*V*_p_ (cm^3^ g^−1^)	*D*_p_ (nm)
APO	17.90	0.055	1.25
m-APO/C_60_(5)	55.461	0.212	1.26
m-APO/C_60_(10)	54.373	0.207	1.26
m-APO/C_60_(20)	48.873	0.193	1.26

a *S*_BET_: BET surface area. *V*_p_: total pore volume. *D*_p_: average pore diameter calculated using the BJH method.

**Fig. 9:**
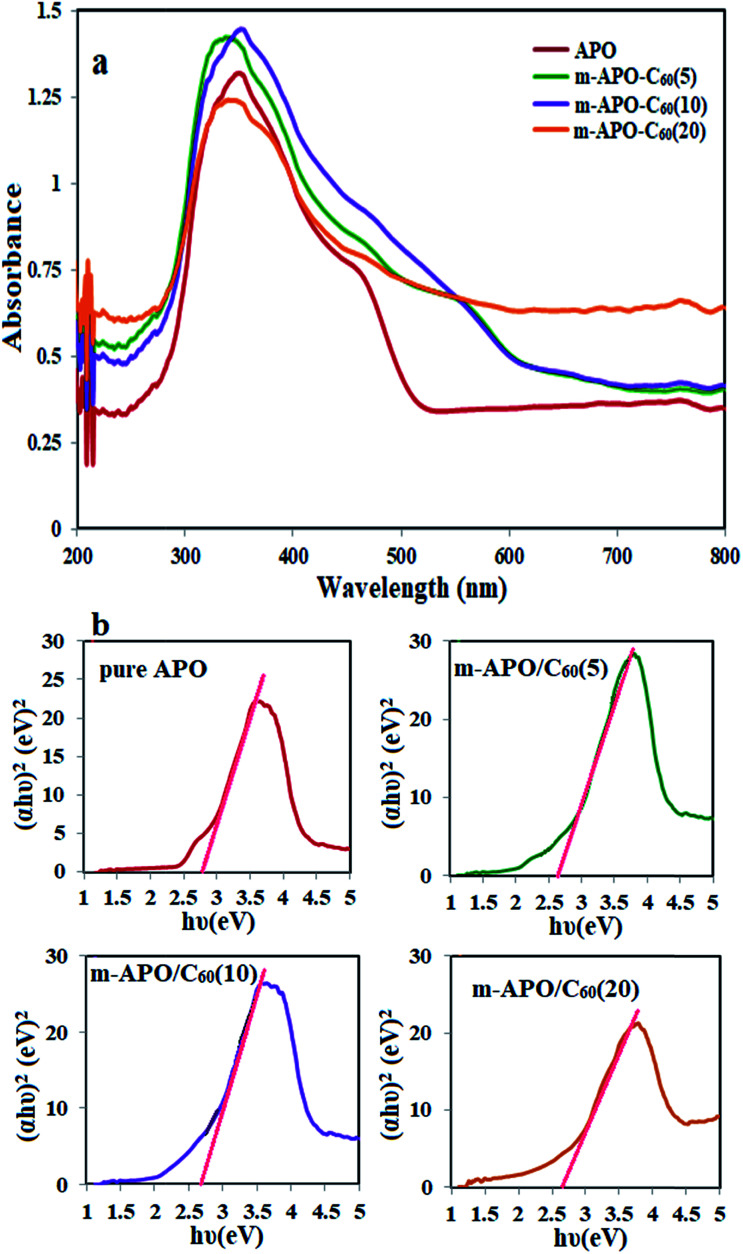
(a) The UV-Vis diffuse reflectance spectra and (b) (*αhν*)^2^−(*hν*) curves for pure APO and the m-APO/C_60_(5), m-APO/C_60_(10) and m-APO/C_60_(20) nanocomposites.

Fig. 9 shows the UV-Vis diffuse reflectance absorption spectra (DRS) of single APO and m-APO/C_60_ nanocomposites with different C_60_ mass ratios. It can be seen in Fig. 9(a) that APO and the m-APO/C_60_ nanocomposites show strong absorption bands above 300 nm, which are assigned to the intrinsic band gap absorption of APO. Compared to APO, the m-APO/C_60_ nanocomposites show more intensive absorption over the whole visible light region, consistent with the gray color of the samples, relating to the presence of C_60_ molecules. The band gap energy (*E*_g_) of the samples can be obtained from the following formula: (*αhν*)^1/2^ = *B*(*hν* − *E*_g_), where *α*, *ν* and *B* are the absorption coefficient, light frequency and proportionality constant, respectively. As shown in Fig. 9(b), the value of *hν* extrapolated to *α* = 0 gives the band gap energy. Based on UV-DRS spectroscopy studies, the band gaps were estimated to be 2.75 eV, 2.60 eV, 2.72 eV and 2.70 eV for pure APO, m-APO/C_60_(5), m-APO/C_60_(10) and m-APO/C_60_(20), respectively. Among them, the m-APO/C_60_(5) nanocomposite had lower band gaps.

### 3.2. Photocatalytic activity

**Fig. 10:**
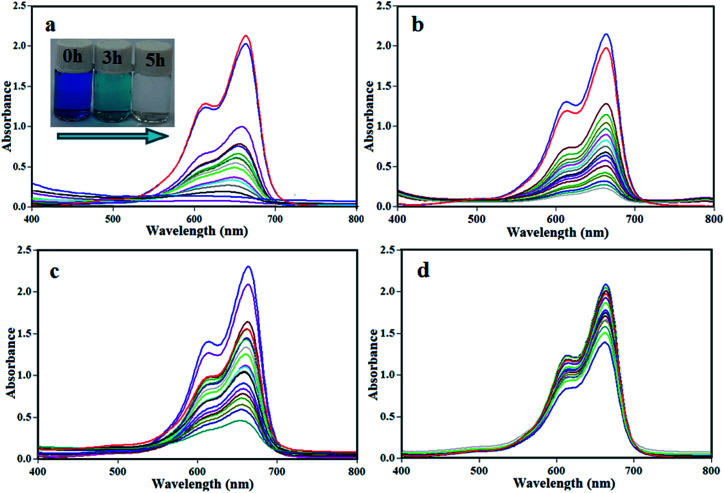
UV-Vis spectral changes of MB aqueous solutions over different catalysts: (a) m-APO/C_60_(5), (b) m-APO/C_60_(10), (c) m-APO/C_60_(20) and (d) pure APO under visible light. The inset photo in (a) shows the color change of the MB solution during photodegradation. Experimental conditions: [MB] = 25 mg L^−1^, [catalyst] = 0.05 g and [H_2_O_2_] = 30 wt%, 0.5 mL under visible light irradiation for 5 h.

**Fig. 11:**
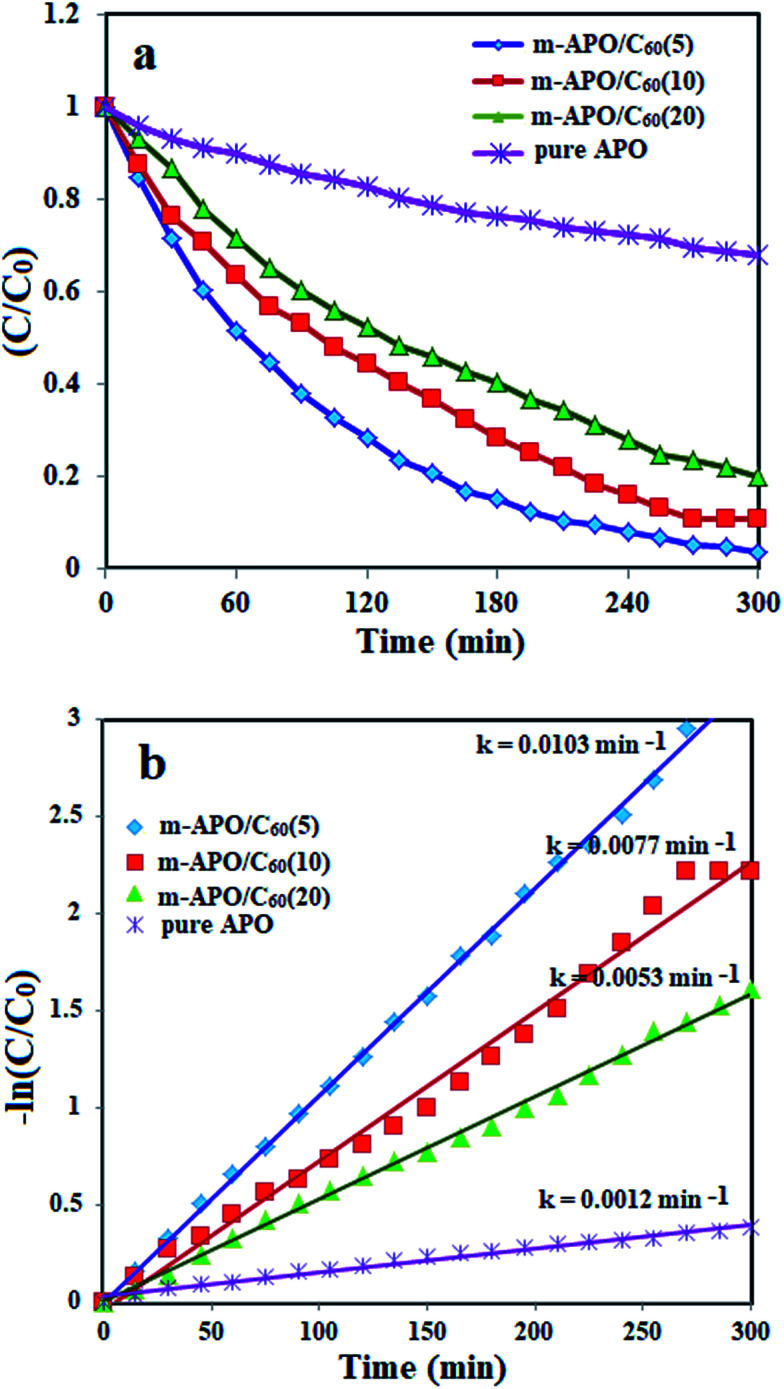
(a) Concentration changes (*C*/*C*_0_) and (b) plot of −ln(*C*/*C*_0_) *versus* irradiation time of MB dye over APO/C_60_(5), m-APO/C_60_(10), m-APO/C_60_(20) and pure APO photocatalysts. Experimental conditions: [MB] = 25 mg L^−1^, [catalyst] = 0.05 g and [H_2_O_2_] = 30 wt%, 0.5 mL under visible light irradiation for 5 h.

The photocatalytic degradation of organic contaminants has attracted considerable attention because of its potential to solve serious environmental difficulties such as aquatic pollution and printing/textile wastewater, and the associated toxicity and perturbation to aquatic life. Various photocatalysts have been successfully developed for environmental remediation.^60^ Among them, TiO_2_ is one of the best, due to its non-toxicity, suitable stability and high photocatalytic activity.^61^ However, TiO_2_ is responsive only to UV light, which accounts for no more than 4% of the solar spectrum, greatly limiting its photocatalytic efficiency and suitable applications. Developing a novel photocatalyst with efficient visible light absorption and excellent stability remains a great challenge. The m-APO/C_60_ nanocomposites prepared in this study can be an appropriate candidate. The photocatalytic activity of the m-APO/C_60_ nanocomposites was evaluated by the degradation of methylene blue (MB) dye in aqueous solution under visible light irradiation and at room temperature. The UV-Vis spectral changes of the MB aqueous solution over the m-APO/C_60_(5) photocatalyst is plotted in Fig. 10 as a function of irradiation time. The degradation percentage was calculated using the equation [(*C*_0_ − *C*)/*C*_0_] × 100%, where *C* is the concentration of the reactant after irradiation at time *t* and *C*_0_ is the concentration of the MB dye after adsorption–desorption equilibrium. Fig. 10 shows that the intensity of the maximum absorption peak of MB at 663 nm decreases intensely as time increases and approximately disappears within 300 min. For comparison purposes, we additionally performed the experiments on the degradation of MB with m-APO/C_60_(5), m-APO/C_60_(10), m-APO/C_60_(20) and pure APO under identical experimental conditions. The degradation efficiencies (%) of these photocatalysts were found to be 95%, 89%, 80% and 33%, respectively, within 300 min under visible light irradiation. To determine the photocatalytic degradation kinetics of MB degradation, the pseudo first order model was used: ln(*C*_0_/*C*) = *kt*, where *C*_0_ and *C* are the dye concentrations before and after visible light irradiation, respectively, *k* is the pseudo first order rate constant, and *t* is the reaction time. As shown in Fig. 11, the *k* values for the degradation of MB over the m-APO/C_60_(5), m-APO/C_60_(10), m-APO/C_60_(20) and pure APO catalysts were determined to be 1.03 × 10^−2^, 7.70 × 10^−3^, 5.30 × 10^−3^ and 1.20 × 10^−3^ min^−1^, respectively. The results indicate that the photocatalytic activity of APO could be improved by the incorporation of Fe_3_O_4_ nanoparticles and C_60_ molecules. From the photocatalytic activity results, it can be further confirmed that the low optimal molar ratio of m-Ag_3_PO_4_/C_60_ was good for increasing surface active sites and photocatalytic performance.

**Fig. 12:**
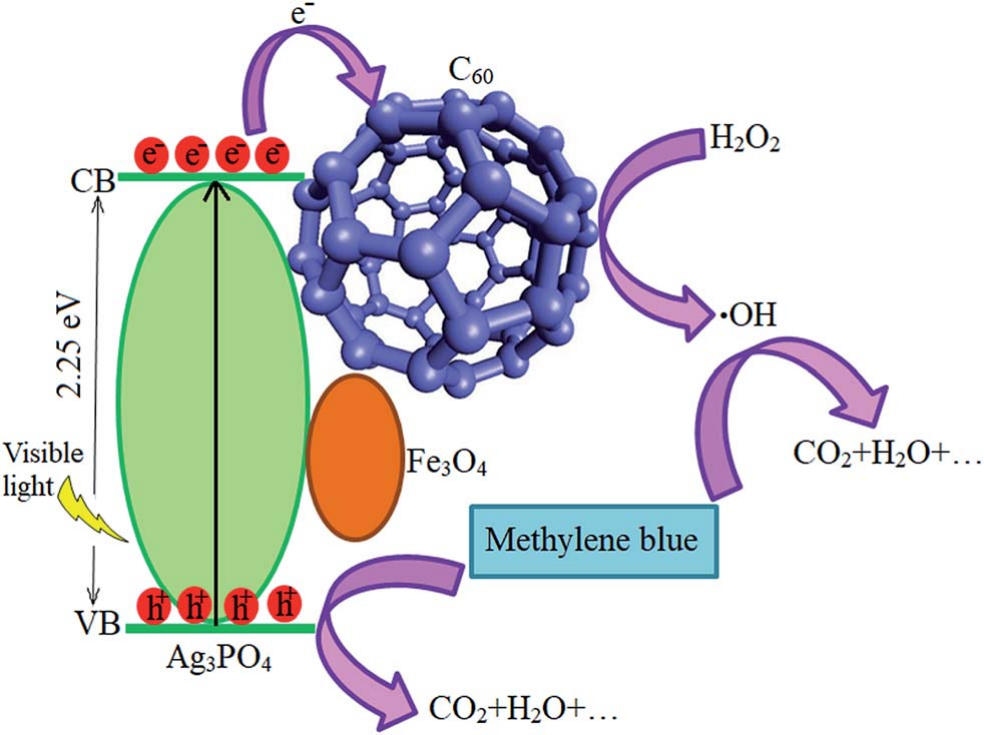
A proposed mechanism for the photocatalytic degradation of MB over the m-APO/C_60_ nanocomposite.

A possible mechanism for the photocatalytic degradation of MB dye is proposed as follows. The generated light with an appropriate wavelength could excite APO to produce photogenerated electrons and holes. Then, the photogenerated electrons are transferred to the surface of the C_60_ particles, which act as effective electron acceptors.^62^ C_60_ is a conjugated structure in which the charge carriers act as massless fermions, leading to unique charge transfer properties. Therefore, the photogenerated electrons of APO could transfer easily from the CB band to the C_60_ particles. As a result of the extreme inhibition of the photogenerated electrons and holes, the photocatalytic activity is enhanced. The electrons on the surface of C_60_ can react with the dissolved H_2_O_2_ to produce hydroxyl radicals, while the holes are scavenged by the adsorbed water or OH^−^ to form ˙OH radicals. Finally, these active species could oxidize the MB molecules adsorbed on the active sites of m-APO/C_60_ through electrostatic attraction or π–π stacking, resulting in dye degradation and production of CO_2_, H_2_O, *etc.*, as acquitted materials (eqn (1)–(4)).^63^ According to this, a schematic representation of the proposed mechanism is illustrated in Fig. 12.

1 Ag_3_PO_4_/Fe_3_O_4_ + visible light → e_CB_^−^ + h_VB_^+^

2 C_60_ + e_CB_^−^ → C_60_˙^−^

3 h_VB_^+^ + H_2_O/OH^−^ → ˙OH + H^+^

4 C_60_˙^−^ + H_2_O_2_ → ˙OH + OH^−^

5 MB + ˙OH and h_VB_^+^ → CO_2_ + H_2_O + …

### 3.3. Catalytic activity

**Fig. 13:**
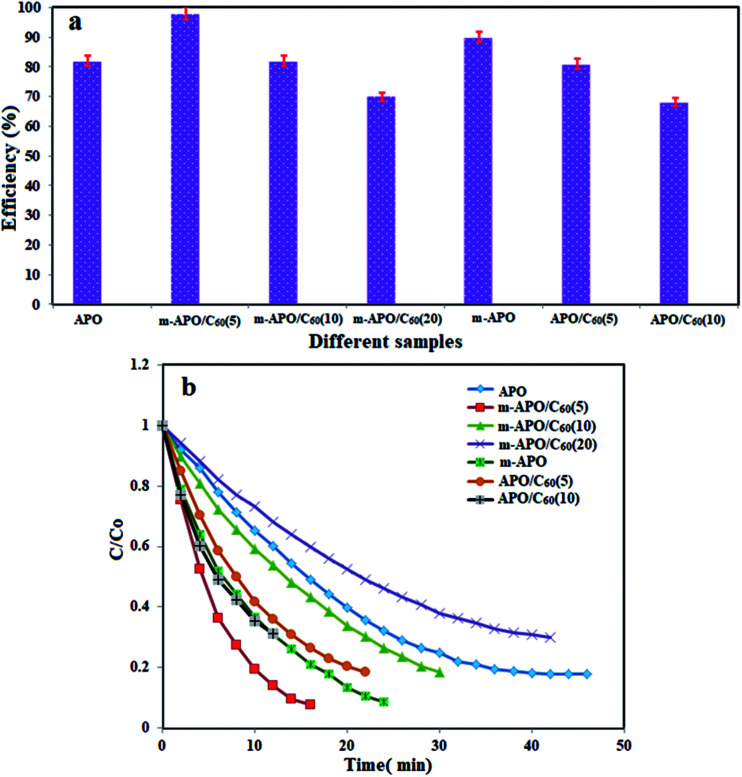
(a) Comparison of the reduction efficiencies of 4-NP with NaBH_4_ over different catalysts, and (b) concentration changes (*C*/*C*_0_) of 4-NP as a function of time. Experimental conditions: [4-NP] = 0.2 mM, [catalyst] = 2.5 mg and [NaBH_4_] = 20 mM, 0.5 ml at 25 °C.

It is necessary to develop environmentally friendly and clean techniques to remove pollutants such as nitrophenols and their derivatives from industrial wastewater. To evaluate the catalytic activity of the prepared nanocomposites in this research, the reduction of nitrophenols (4-NP) by excess NaBH_4_ was used as the model pollutant in aqueous solution. The catalytic process was monitored by UV-Vis spectroscopy. This showed the concentration changes (*C*/*C*_0_) and efficiencies of 4-NP reduction in the presence of different samples. Among these, the samples containing the prepared nanocomposite catalysts exhibited the best catalytic performance (Fig. 13).

**Fig. 14:**
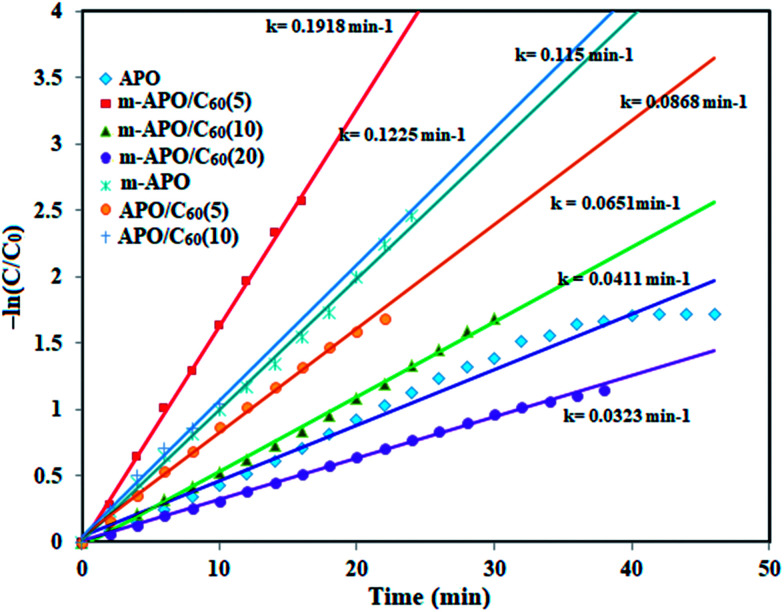
The plot of −ln(*C*/*C*_0_) *versus* reduction time for the reduction of 4-NP in the presence of the different catalysts. Experimental conditions: [4-NP] = 0.2 mM, [catalyst] = 2.5 mg and [NaBH_4_] = 20 mM, 0.5 ml at 25 °C.

In this reduction process, the overall concentration of NaBH_4_ was 20 mM while the overall concentration of 4-NP was 0.2 mM. Considering the higher concentration of NaBH_4_ compared to that of 4-NP, it is logical to assume that the concentration of BH_4_^−^ remains constant during the reaction. Thus, pseudo first order kinetics could be used to evaluate the kinetics of the catalytic reaction. The absorbance of 4-NP is proportional to its concentration in solution, and the absorbance at time *t* (*A*) and time *t* = 0 (*A*_0_) are equivalent to the concentration at time *t* (*C*) and time *t* = 0 (*C*_0_). The rate constant (*k*) could be obtained from the linear plot of −ln(*C*/*C*_0_) *versus* the reduction time in minutes. As shown in Fig. 14, the −ln(*C*/*C*_0_) *versus* time plots indicate a good linear correlation. The results show that the catalytic activity of the m-APO-C_60_(5) nanocomposite is higher than those of pure APO_4_, modified m-APO/C_60_(10) and m-APO/C_60_(20), m-APO, APO-C_60_(5) and APO/C_60_(10).

**Fig. 15:**
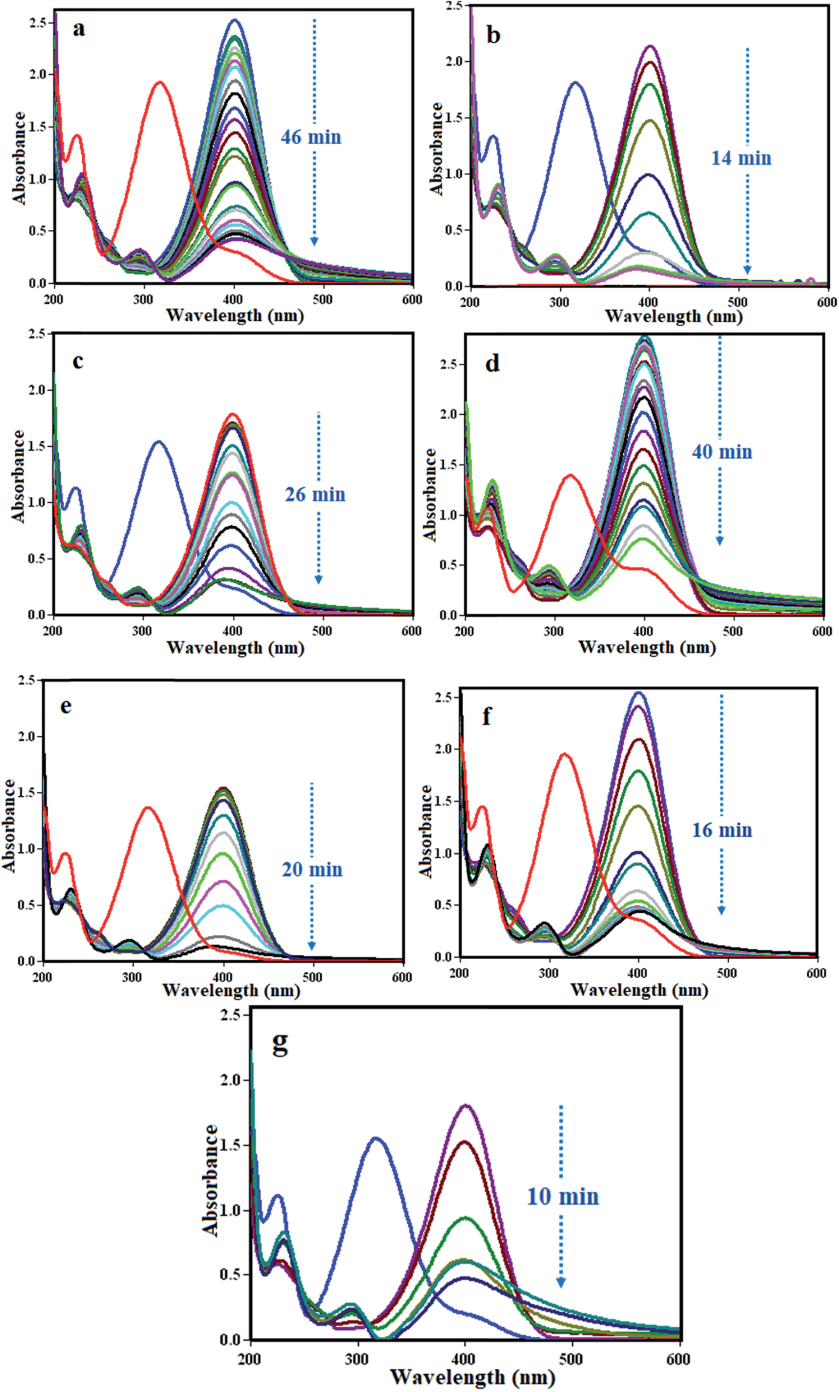
UV-Vis spectral changes during the reduction of 4-NP with NaBH_4_ over different catalysts: (a) pure APO, (b) m-APO-C_60_(5), (c) m-APO-C_60_(10), (d) m-APO-C_60_(20), (e) m-APO, (f) APO-C_60_(5) and (g) APO-C_60_(10). Experimental conditions: [4-NP] = 0.2 mM, [catalyst] = 2.5 mg and [NaBH_4_] = 20 mM, 0.5 ml at 25 °C.

Fig. 15 shows the UV-Vis spectral changes of a 4-NP aqueous solution over different catalyst samples. It can be seen in Fig. 15(b) that the absorption peak of 4-NP undergoes a red shift from 316 to 400 nm immediately after the addition of an aqueous solution of NaBH_4_, corresponding to a significant change in the solution color from light yellow to yellow-green due to the formation of the 4-nitrophenolate ion. In the absence of the m-APO/C_60_(5) nanocomposite catalyst (2.5 mg), the absorption peak at about 400 nm remained unchanged for a long time, indicating that the NaBH_4_ itself could not reduce the 4-nitrophenolate ion without a catalyst. In the presence of the m-APO-C_60_(5) nanocomposite catalyst and NaBH_4_, 4-NP was reduced and the intensity of the absorption peak at about 400 nm decreased progressively as time passed, and after almost 16 min disappeared completely. Meanwhile, a new absorption peak with increasing intensity appeared at about 295 nm. This peak was attributed to the typical absorption of 4-amino phenol (4-AP).

**Fig. 16:**
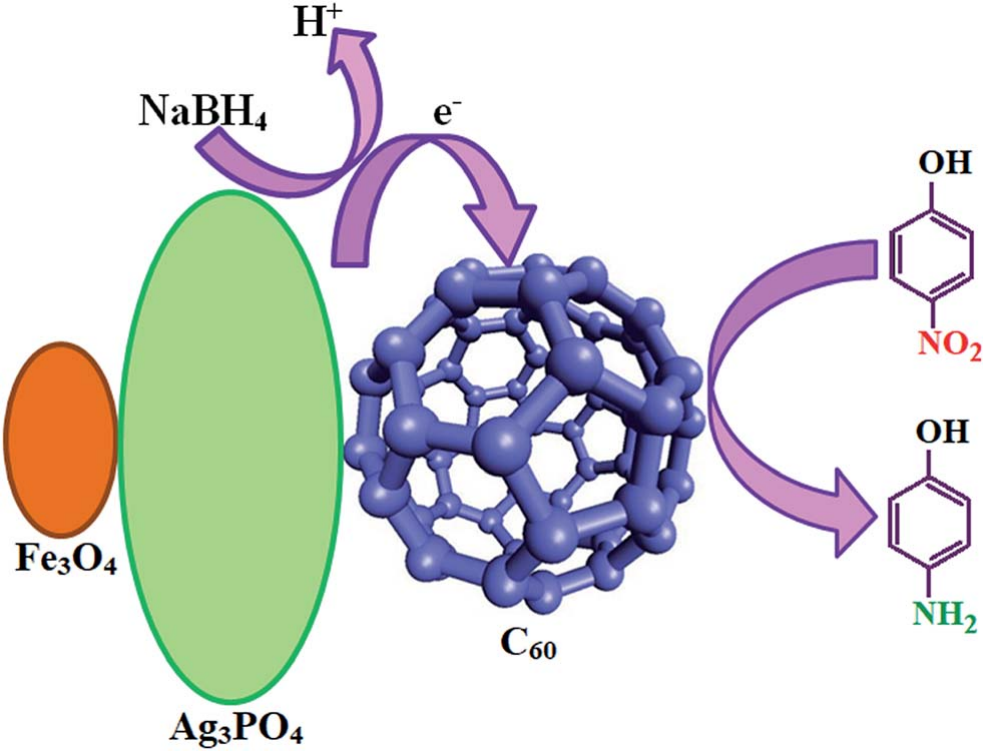
Proposed mechanism for 4-nitrophenol reduction with NaBH_4_ over m-APO-C_60_.

Fig. 16 shows the probable reduction mechanisms of 4-NP to 4-AP in the presence of m-APO/C_60_. According to the results, it was evident that as the m-APO-C_60_ content (>20%) increased, the catalytic activity deteriorated, highlighting the important role of the loading percentage and intimate contact between C_60_ and Ag_3_PO_4_/Fe_3_O_4_ in determining catalytic efficiency. This means that with higher amounts of C_60_, the number of active catalytic reaction sites decreases, causing a negative influence on the catalytic processes. The excessive amount of C_60_ may cover the active sites at the Ag_3_PO_4_/Fe_3_O_4_ surface and also hinder the contact with 4-NP. Furthermore, from the catalytic activity results, it can be concluded that the low optimal molar ratio of m-APO/C_60_ is conducive to increasing the number of active surface sites and enhancing catalytic performance. The highest catalytic performance of the m-APO/C_60_ composite can be attributed to the intimate contact between Ag_3_PO_4_/Fe_3_O_4_ and C_60_, which facilitates electron transfer. A probable mechanism for the reduction of 4-NP to 4-AP in the presence of m-APO-C_60_ is shown in Fig. 16.

**Fig. 17:**
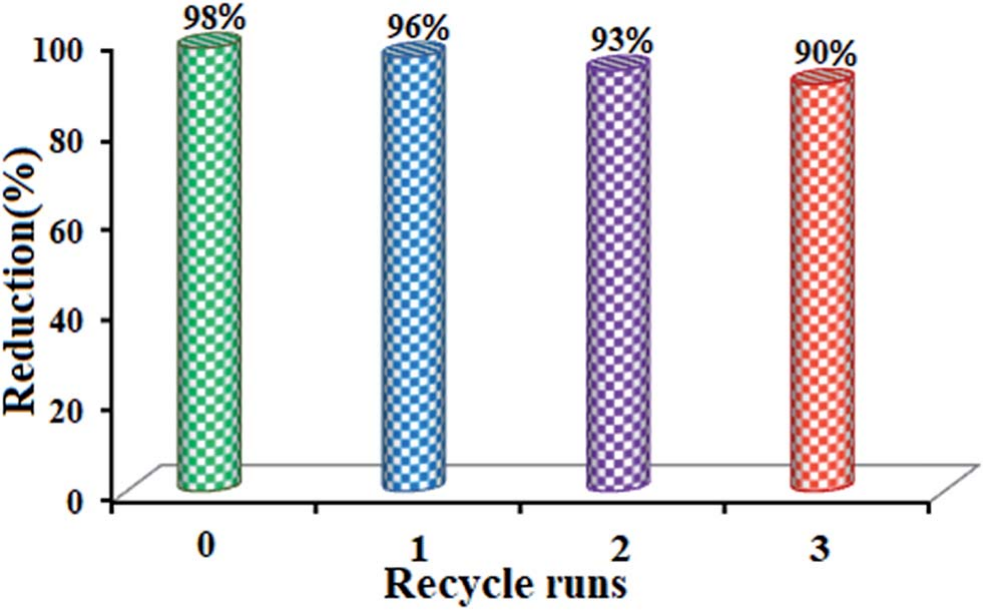
Recycling experiments for the m-APO-C_60_(5) nanocomposite in the catalytic reduction of 4-NP.

Moreover, the presence of Fe_3_O_4_ in the composites caused them to be magnetically separable during the catalytic reactions. Recyclability is a very important parameter to assess how practical and reusable the catalyst is. Therefore, the recovery and reusability of the m-APO-C_60_(5) catalyst was determined for the reduction of 4-NP under the present reaction conditions. After the reaction was completed, the m-APO/C_60_(5) nanocomposite was separated from the reaction mixture by an external magnet. The catalyst was washed with water and ethanol several times, then dried and reused for the next reaction. Three consecutive catalyst recoveries were performed showing a good catalytic activity (Fig. 17).

**Fig. 18:**
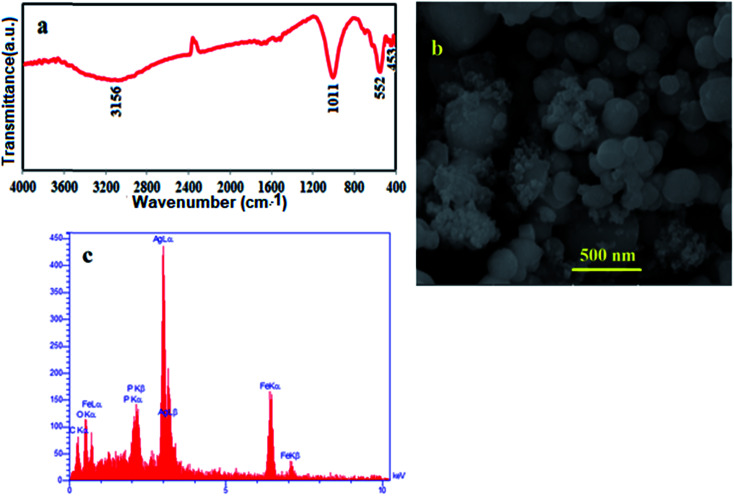
(a) FT-IR spectrum, (b) SEM image and (c) EDX analysis of the recycled m-APO/C_60_(5) catalyst.

For up to three catalytic cycles, no significant loss in activity was observed, indicating that the as-prepared catalyst was stable and efficient for the reduction of nitro compounds. As observed in Fig. 18, the FT-IR spectrum, SEM image and EDX map of the recycled catalyst do not show any significant change after the third run, in comparison with those of the fresh catalyst (see Fig. 2, 4 and 5). This observation confirms that the m-APO/C_60_(5) nanocomposite is stable under the reaction conditions and is not affected by the reactants.

### 3.4. Antibacterial activity

The antibacterial activity of the samples was analyzed against four bacteria, including *Bacillus cereus*, *Staphylococcus aureus*, *Klebsiella pneumonia* and *Escherichia coli* using the disk diffusion method. The results of the antibacterial activity tests for pure APO, m-APO/C_60_(5), m-APO/C_60_(10) and m-APO/C_60_(20) are shown in Table 3. The results show that the m-APO/C_60_(5) nanocomposite has a relatively good antibacterial activity compared to pure APO, *i.e.* the bacteria cells were killed at a concentration of 10 mg ml^−1^. The highest activity was obtained for the m-APO/C_60_(5) nanocomposite against *B. cereus*, while the lowest activity was observed for m-APO/C_60_(5) against *E. coli*. The biosynthesized m-APO/C_60_(5) nanocomposite exhibited a greater antimicrobial activity towards Gram-positive microorganisms than Gram-negative ones. The results showing the antibacterial activity of the m-APO/C_60_(5) nanocomposite with different concentrations are presented in Table 4, indicating that the m-APO/C_60_(5) nanocomposite has different antibacterial activity with different concentrations. It also represents the inhibition zone of these bacteria. The highest activity was obtained for the m-APO/C_60_(5) (1.25 mg ml^−1^) nanocomposite against *S. aureus*, while the lowest activity was observed for the m-APO/C_60_(5) (0.625 mg ml^−1^) nanocomposite against *K. pneumonia* and *B. cereus*. The potential antimicrobial activities presented by the m-APO/C_60_(5) nanocomposite have made it a promising candidate for a novel generation of antimicrobials. The clear mechanism of the m-APO/C_60_(5) nanocomposite’s interaction with bacteria is not well known. However, several main mechanisms underpin the biocidal properties of the m-APO/C_60_(5) nanocomposite against microorganisms. Firstly, the m-APO/C_60_(5) nanocomposite attaches to the negatively charged cell surface, altering the physical and chemical properties of the cell membrane and the cell wall, and disturbing important functions such as permeability, osmoregulation, electron transport and respiration.^64^ Secondly, the m-APO/C_60_(5) nanocomposite can cause further damage to bacterial cells by permeating the cell and interacting with DNA, proteins and other phosphorus- and sulfur-containing cell constituents.^65^ Thirdly, the m-APO/C_60_(5) nanocomposite releases silver ions, generating an amplified biocidal effect which is size- and dose-dependent.^66^

**Table 3:** Average size of the inhibition zones for pure APO and the synthesized m-APO/C_60_ nanocomposites

Bacteria	Type	Pure APO	m-APO/C_60_(5)	m-APO/C_60_(10)	m-APO/C_60_(20)	Disc standard
*E. coli*	Gram-negative	8	9	9	8	27
*K. pneumonia*	Gram-negative	9	10	9	9	25
*S. aureus*	Gram-positive	8	10	10	10	26
*B. cereus*	Gram-positive	8	12	10	9	16

**Table 4:** Average size of the inhibition zones for pure APO and the m-APO/C_60_(5) nanocomposite with different concentrations

Sample	Bacteria	5 mg ml^−1^	2.5 mg ml^−1^	1.25 mg ml^−1^	0.625 mg ml^−1^	0.312 mg ml^−1^	Disc standard
m-APO/C_60_(5) nanocomposite	*E. coli*	11	9	11	9	9	25
	*K. pneumonia*	8	11	8	7	9	27
	*S. aureus*	11	12	16	9	8	30
	*B. cereus*	8	10	7	7	7	15
Pure APO	*E. coli*	8	8	8	7	0	22
	*K. pneumonia*	8	7	7	7	7	24
	*S. aureus*	9	9	8	8	7	25
	*B. cereus*	7	7	7	7	0	25

## 4. Conclusions

In this study, m-APO/C_60_ nanocomposites were synthesized using a facile and effective hydrothermal route. The synthesized m-APO/C_60_ nanocomposites were spherical, 30 nm in size and crystalline in nature, and exhibited absorptions at ∼300–620 nm. The m-APO/C_60_(5) nanocomposite had a band gap of about 2.6 eV. Its photocatalytic activity was much higher than that of pure Ag_3_PO_4_ or those of the other nanocomposites for degrading methylene blue. The results showed a 95% degradation of methylene blue (MB) (25 mg L^−1^) within 5 h in the presence of the Ag_3_PO_4_/Fe_3_O_4_/C_60_ nanocomposite and H_2_O_2_. In addition, this new nanocomposite showed an 98% reduction of 4-nitrophenol (4-NP) (0.2 mM) with excess NaBH_4_. The formed m-APO/C_60_ nanocomposites were quite stable, showed good antibacterial activity and were utilized as catalysts for the reduction of several aromatic nitro compounds (98% reduction of 4-NP) into their corresponding amino derivatives. It is expected that this kind of m-APO/C_60_ nanocomposite would provide new insights for the design and construction of high performance photocatalysts for eliminating environmental pollution damage. This nanocomposite can easily be removed using an external magnet and prevents the secondary pollution of water. The nanocomposites are therefore both economically and environmentally friendly, and they could be a good option for eliminating water contaminants.

## Conflicts of interest

There are no conflicts of interest to declare.

## Supplementary Material
